# Implementing an adapted patient safety bundle for pregnancy-related severe hypertension to improve recognition, response, and respectful care in the outpatient setting

**DOI:** 10.1186/s43058-026-01012-y

**Published:** 2026-06-22

**Authors:** E. Nicole Teal, Narges Farahi, Jennifer Leeman, Feng-Chang Lin, Kimberly Harper, Alexandra F. Lightfoot, M. Kathryn Menard

**Affiliations:** 1 Division of Maternal-Fetal Medicine, Women’s Reproductive Health Research (WRHR) Scholar, Department of Obstetrics, Gynecology and Reproductive Sciences, University of California, 9300 Campus Point Drive, MC 7433, San Diego, CA 92037 USA; 2 Department of Family Medicine, University of North Carolina at Chapel Hill, Chapel Hill, NC USA; 3 School of Nursing, University of North Carolina at Chapel Hill, Chapel Hill, NC USA; 4 Department of Biostatistics, University of North Carolina at Chapel Hill, Chapel Hill, NC USA; 5 Collaborative for Maternal and Infant Health, University of North Carolina at Chapel Hill, Chapel Hill, NC USA; 6 Department of Health Behavior, Gillings School of Global Public Health, University of North Carolina at Chapel Hill, Chapel Hill, NC USA; 7 Department of Obstetrics and Gynecology, University of North Carolina at Chapel Hill, Chapel Hill, NC USA

**Keywords:** Pregnancy-related hypertension, Severe hypertension, Hypertension bundle, Obstetrics, Implementation science, Community-engagement, Simulation, Respectful care

## Abstract

**Background:**

Severe hypertension (HTN) during pregnancy is highly associated with adverse perinatal outcomes, yet many cases occur in outpatient settings where standardized management is lacking. We used a community-engaged approach to adapt an evidence-based inpatient HTN bundle for outpatient care, creating the Outpatient HTN (O-HTN) Bundle. This study aimed to evaluate the feasibility, acceptability, and impact of three implementation strategies (1) identifying and preparing champions, (2) providing ongoing training, and (3) simulation.

**Methods:**

We conducted a pre-post pilot study in three Federally Qualified Health Centers (FQHCs) from 9/2021-6/2022. Multidisciplinary clinical teams comprised of a medical assistant, nurse, provider (midwife or physician), and pharmacy staff received training on the O-HTN Bundle and participated in pre- and post-training simulations. Simulations involved a patient actor presenting with severe HTN during a prenatal visit. Trained observers used a checklist to evaluate clinical team fidelity to the O-HTN Bundle algorithm. Observers, clinical team members, and the patient actor assessed provision of respectful care via a Respectful Care Reflection Tool. The primary outcome was fidelity to the O-HTN Bundle; secondary outcomes included respectful care and feasibility and acceptability of the implementation strategies. Feasibility was defined by engagement in trainings/simulations. Acceptability was assessed with a study-specific survey. Paired t-tests compared fidelity and respectful care scores pre- and post-training. Descriptive statistics summarized acceptability and feasibility data.

**Results:**

The implementation strategies were feasible; 19 clinical teams participated, representing 59% of all prenatal providers across the three FQHCs. All 19 teams participated in the O-HTN Bundle training and pre- and post-training simulations. The strategies were also effective: mean fidelity scores improved from 78% pre-training to 95% post-training (*p* = 0.006). Significant gains were made on severe HTN identification, treatment, and escalation to higher level of care. Respectful care scores also improved, though not significantly. Survey results indicated acceptability of the training and simulation model.

**Conclusion:**

In this pilot study, clinic champions, training, and simulation were feasible, acceptable, and effective strategies to improve fidelity to the O-HTN Bundle and thus response to severe HTN in pregnancy. These findings support broader implementation of the O-HTN Bundle.

**Supplementary Information:**

The online version contains supplementary material available at https://doi.org/10.1186/s43058-026-01012-y.

**Table:** 

**Contributions to the literature**
This study:
• Demonstrates the feasibility and acceptability of using champions, training, and simulation as implementation strategies for a severe hypertension in pregnancy safety bundle.
• Illustrates the use of simulation as a strategy for implementing evidence-based guidelines for rare events in the outpatient setting.
• Extends prior inpatient-focused implementation work by implementing an adaptation of the AIM Severe HTN Bundle in outpatient clinics.
• Contributes novel methodology by integrating fidelity assessment and simulation-based evaluation in outpatient obstetric health care.
• Supports the potential for broader dissemination and scale-up of outpatient obstetric patient safety bundles through structured team-based training.

## Introduction

Hypertensive disorders of pregnancy affect 13 to 16% of pregnancies in the United States (US) [1]. They are responsible for 6% of all pregnancy related deaths in the US and an even larger proportion of pregnancy related morbidity [2, 3]. They are also a major contributor to adverse fetal and neonatal outcomes and are associated with increased lifetime risk of cardiovascular disease for the birthing person [4–6]. Furthermore, there are stark disparities in rates of hypertensive disorders of pregnancy; birthing people who are Black, publicly insured, and/or rural dwelling have the highest risk and may have more severe presentations with worse outcomes [7–10]. Hypertension in pregnancy is defined as systolic blood pressure 140 mmHg or greater and/or diastolic blood pressure 90 or greater [11]. Severe hypertension in pregnancy, defined as systolic blood pressure 160 mmHg or greater and/or diastolic blood pressure 110 or greater, has the highest association with adverse outcomes including stroke and death [12, 13]. As such, prompt identification and treatment of severe hypertension is essential to preventing maternal mortality and morbidity and decreasing disparities therein [14].

In 2015, the Alliance for Innovation on Maternal Health (AIM) developed the Severe Hypertension During Pregnancy and Postpartum Period Safety Bundle (HTN Bundle) with a goal of improving early detection and treatment; it was later revised in 2022 [15]. The bundle includes the following five components: (1) Readiness, (2) Recognition, (3) Response, (4) Reporting and Systems Learning, and (5) Respectful, Equitable and Supportive Care [15]. The HTN Bundle has been implemented in hospitals across the country by statewide perinatal quality collaboratives with excellent results. The Illinois Perinatal Quality Collaborative engaged 110 hospitals in quality improvement efforts through its Severe Maternal Hypertension Initiative, which was based on the AIM HTN Bundle. Over the course of the initiative, timely treatment of severe hypertension within 60 minutes improved from 41.5% to 78.9% and severe maternal morbidity (SMM) was reduced at the state population level [16, 17]. Similarly, the California Maternal Quality Care Collaborative implemented a severe hypertension toolkit based on the AIM Bundle across 23 academic and community hospitals in California. During the 18-month initiative, adherence to treatment recommendations increased from 50% to more than 90%, rates of eclampsia fell by 42.6%, and SMM prevalence declined by nearly 20% [18]. The AIM HTN Bundle is now a quality standard for hospitals accredited by The Joint Commission. However, many episodes of severe hypertension are first detected in the outpatient setting where no standardized approach to management has been delineated. Given this, we used a community-engaged approach to adapt the AIM inpatient HTN Bundle for the outpatient setting (O-HTN Bundle) and tailor implementation strategies to support its use. The clinic-facing implementation strategies utilized were clinic champions, training, and simulation. The primary purpose of this pilot study was to assess the effect of these implementation strategies on fidelity to the O-HTN Bundle. Secondarily, we also assessed feasibility and acceptability of the implementation strategies as well as the provision of respectful care.

## Materials and Methods

### Design

We conducted a 9-month, single-arm, pre-post feasibility pilot in three Federally Qualified Health Centers (FQHCs). The primary outcome was clinical team fidelity to the O-HTN Bundle algorithm during a simulated episode of severe HTN in pregnancy. Secondary outcomes included feasibility and acceptability of the implementation strategies and the provision of respectful care in the simulation. The study was approved as a quality improvement study by the University of North Carolina at Chapel Hill Institutional Review Board.

### Settings and population

The study was conducted at three FQHCs in North Carolina who are all part of a health system called Piedmont Health Services (PHS). PHS is a multi-site FQHC organization serving a multi-county service area in north-central North Carolina. PHS is the largest FQHC provider of perinatal care in North Carolina and serves a large population of minoritized patients. In 2021, PHS provided prenatal care to 1,041 individuals with the following racial/ethnic breakdown: 77% Hispanic (of any race), 8% non-Hispanic Black/African American; 9% non-Hispanic White; 3% unknown race; 2% Asian; and <1% multiracial. The majority of Hispanic prenatal patients at PHS prefer care in Spanish. Fifty-five percent of patients in 2021 were uninsured during their pregnancies following a period of Medicaid presumptive eligibility (a large percentage are deemed Medicaid ineligible by virtue of citizenship status), while 37% had Medicaid and 8% were privately insured.

### The intervention: O-HTN Bundle

Table 1 provides an overview of components of the AIM Inpatient Severe HTN in Pregnancy Patient Safety Bundle and how they were adapted to create the O-HTN Bundle. The community-engaged approach used to adapt the intervention was guided by the Framework for Reporting Adaptations and Modifications- Expanded (FRAME) [19] and led by our coalition of partners, including patients with lived experience of hypertension in pregnancy, clinic providers, staff and champions, and broader community partners who support birthing mothers. The full adaptation process is described elsewhere [20].

**Table 1:** Original HTN Bundle and adapted O-HTN Bundle components [15]

HTN Bundle Component	Elements of HTN Bundle	Adapted for O-HTN Bundle
Readiness	Develop (1) a standard protocol for maternal early warning signs, diagnostic criteria, monitoring and treatment of severe preeclampsia/eclampsia; (2) a process for the timely triage and evaluation of pregnant and postpartum patients with severe hypertension or related symptoms; and (3) a system plan for escalation, obtaining appropriate consultation, and maternal transfer as needed	**O-HTN Algorithm:** A protocol detailing recommended management of episodes of severe hypertension adapted to the context of each individual clinic (Fig. 1)
	Ensure rapid access to medications used for severe hypertension/eclampsia	If clinic has a pharmacy, get nifedipine 10 mg IR on formulary and develop a process for ordering and administering it in an emergency
Recognition	Ensure accurate measurement and assessment of blood pressure for every pregnant and postpartum patient	No change
	Provide education to health care team members on the recognition of signs, symptoms, and treatment of severe hypertension	No change
Response	Utilize a standardized protocol with checklists and escalation policies including a standard response to maternal early warning signs, listening and investigating patient-reported and observed symptoms, and assessment of standard labs for the management of patients with severe hypertension or related symptoms	Incorporated in O-HTN Algorithm (Fig. 1)
Respectful Care	Engage in open, transparent, and empathetic communication with pregnant and postpartum people and their identified support network to understand diagnoses, options, and treatment plans	Respectful Care Reflection Tool (Fig. 4)
	Include pregnant and postpartum persons as part of the multidisciplinary care team to establish trust and ensure informed, shared decision-making that incorporates the pregnant and postpartum person’s values and goals

**Fig. 1:**
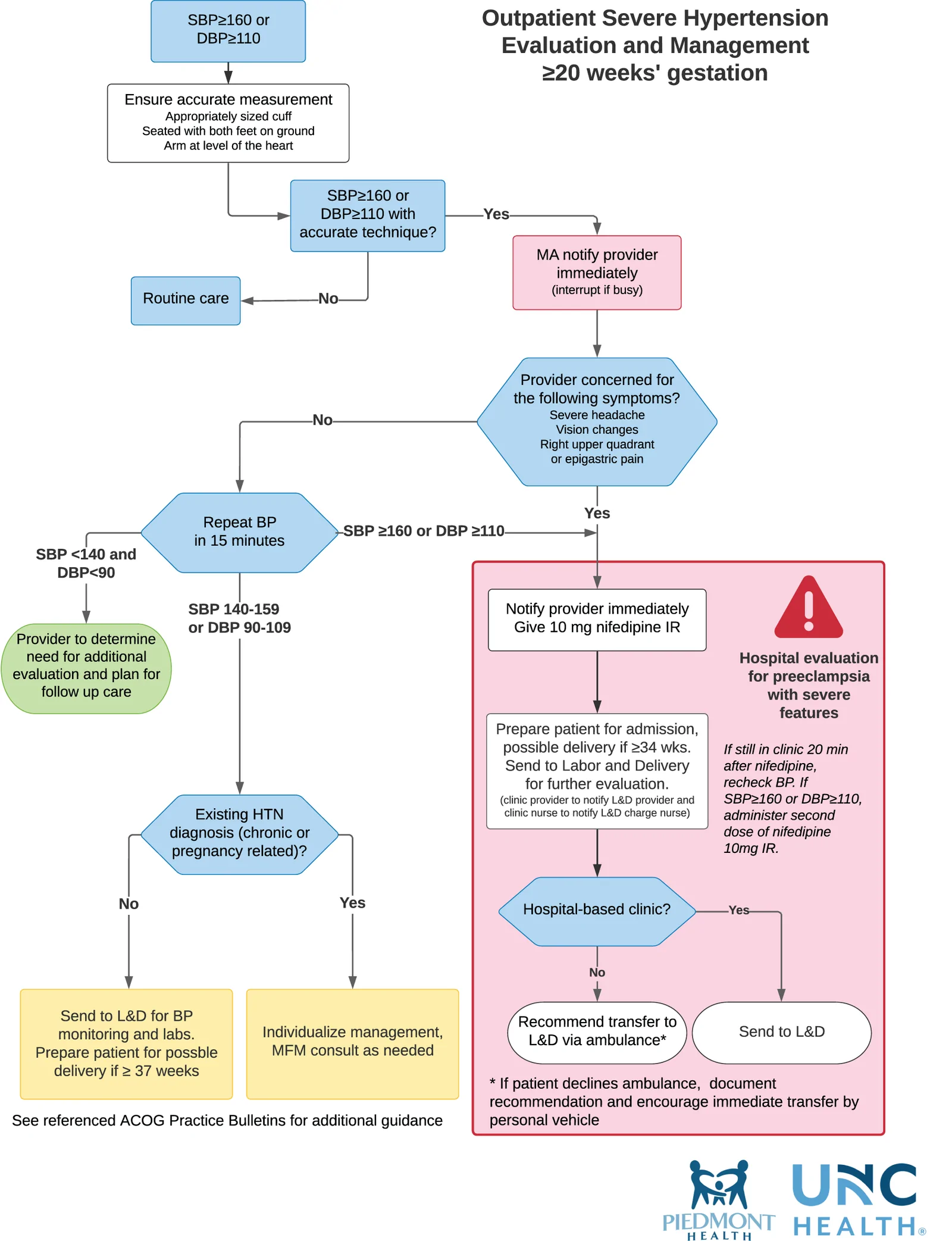
Adapted algorithm for recognition of and response to severe hypertension in pregnancy

The adapted O-HTN Bundle includes: a protocol detailing evidence guidelines for the management of episodes of severe hypertension that each clinic adapts to their context (O-HTN Algorithm), access to medications for emergent treatment of severe HTN in pregnancy, and evidence-based guidelines for accurate blood pressure assessment, recognition of signs and symptoms of HTN, response to severe HTN, and respectful care provision (Table 1).

### Implementation strategies

**Fig. 2:**
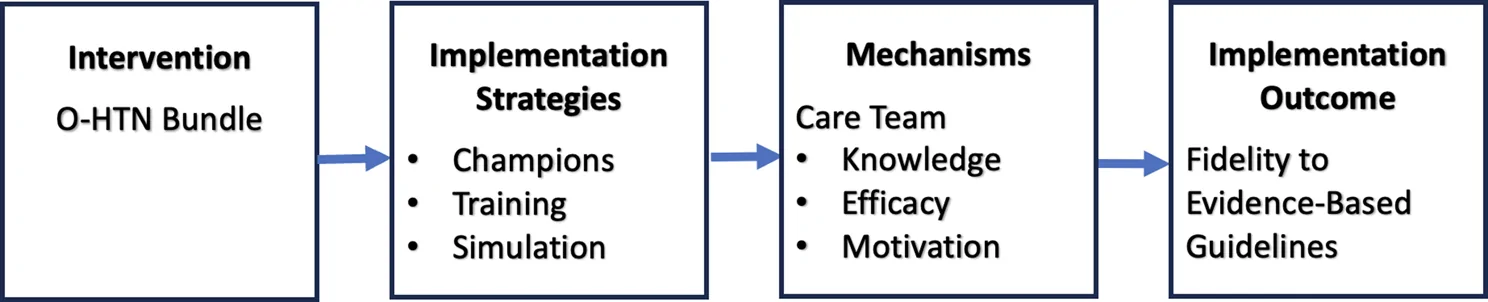
Causal pathway

We engaged our coalition of community and clinic partners to select and tailor implementation strategies. We started by identifying the strategies that clinic partners already used to implement change in practice, with the goal of building on their strengths [21]. Our clinic partners had experience with clinic champions and in-clinic trainings (i.e., lunch and learns). We engaged our community and clinic partners and reviewed the literature to identify the multi-level factors (i.e., determinants) likely to influence implementation of the O-HTN Bundle in the Piedmont clinics. Guided by the Consolidated Framework for Implementation Research (CFIR) [22], factors were identified at the level of innovation recipients (distrust of healthcare system, family commitments), innovation deliverers (quality of communication, knowledge and experience with severe hypertension in pregnancy), inner setting (presence of a pharmacy), and outer setting (distance to hospital). The engagement process used to identify determinants and tailor implementation is reported elsewhere [20, 23]. In the process of tailoring strategies, we identified an additional determinant - severe HTN is a relatively rare occurrence in many outpatient settings, and therefore care teams have limited opportunity to apply what they learn in trainings. To address this barrier we selected simulation as an additional implementation strategy. Simulation allowed for experiential learning and application of individuals’ skills and multidisciplinary team-based communication and care [24]. Table 2 provides an overview of the tailored implementation strategies, named and specified according to terminology from the Expert Recommendations for Implementation Change (ERIC) and described following Proctor et al.’s recommendations [24, 25]. Figure 2 summarizes the causal links between the strategies, their mechanisms of change and intended outcome.

**Table 2:** Implementation strategies and specification

Strategy	Actor	Actions	Action Target	Dose
Identify and prepare champions	Research team members	Identify clinical team members who could champion the work	Clinical team members	1 champion per clinic, ~6 preparation sessions per champion
Provide ongoing training	Clinicians from research team	Provide training to all clinical team members on severe hypertension in pregnancy	Care team member’s individual Knowledge and motivation	1 training session with additional training/feedback following simulations
Simulate change	Clinical team members, patient actors, research team members	Practice responding to a simulated episode of severe hypertension in pregnancy	Care team’s collective efficacy and motivation	2 simulations per clinical team

### Procedures

We identified a provider champion at each site and collaborated with them to provide training and simulation in the three FQHCs from September 2021 to June 2022.

We conducted simulations with the clinic care teams involved in care during pregnancy and post-partum. The clinical teams consisted of a medical assistant, nurse, provider (midwife or physician), and pharmacy staff member; to the extent possible we conducted a simulation with each of the providers as the FQHCs. Patient actors were hired by the research team to participate in the simulations, in the role of a patient presenting for a third trimester prenatal visit. The simulation began with the medical assistant taking the patient’s blood pressure and then informing the provider of the patient’s blood pressure. The care team then had a simulated clinical visit with the patient where they were asked to respond to the situation at hand (simulation guide in Appendix 1). A research team member observed the simulated clinical encounter. At the end of each simulation, simulation facilitators from the research team held a debrief with the clinical team to discuss the team’s performance including what went well and opportunities for improvement.

First, a baseline simulation was conducted with each clinical team at each participating FQHC. Next, clinical teams received training on risks, diagnosis, and management of severe HTN in pregnancy. Training was via a recorded lecture shown to clinical staff at a standing staff meeting at each FHQC and included a heavy focus on recognition of and response to severe HTN and the adapted outpatient severe HTN management algorithm. There was also a section of the training dedicated to discussion of respectful care in general and patient perspectives on the concept gathered through our engagement with patients. Physicians from the research team were present in person at the training to discuss the learnings from the training and answer questions. The training was made available as an online webinar for review by clinic staff who were unable to attend the staff meeting.

One to two weeks following the training, as clinical scheduling allowed, simulation was repeated with each clinical team at each FQHC. The post-training simulation presented an identical clinical scenario as the baseline simulation.

### Measures

**Fig. 3:**
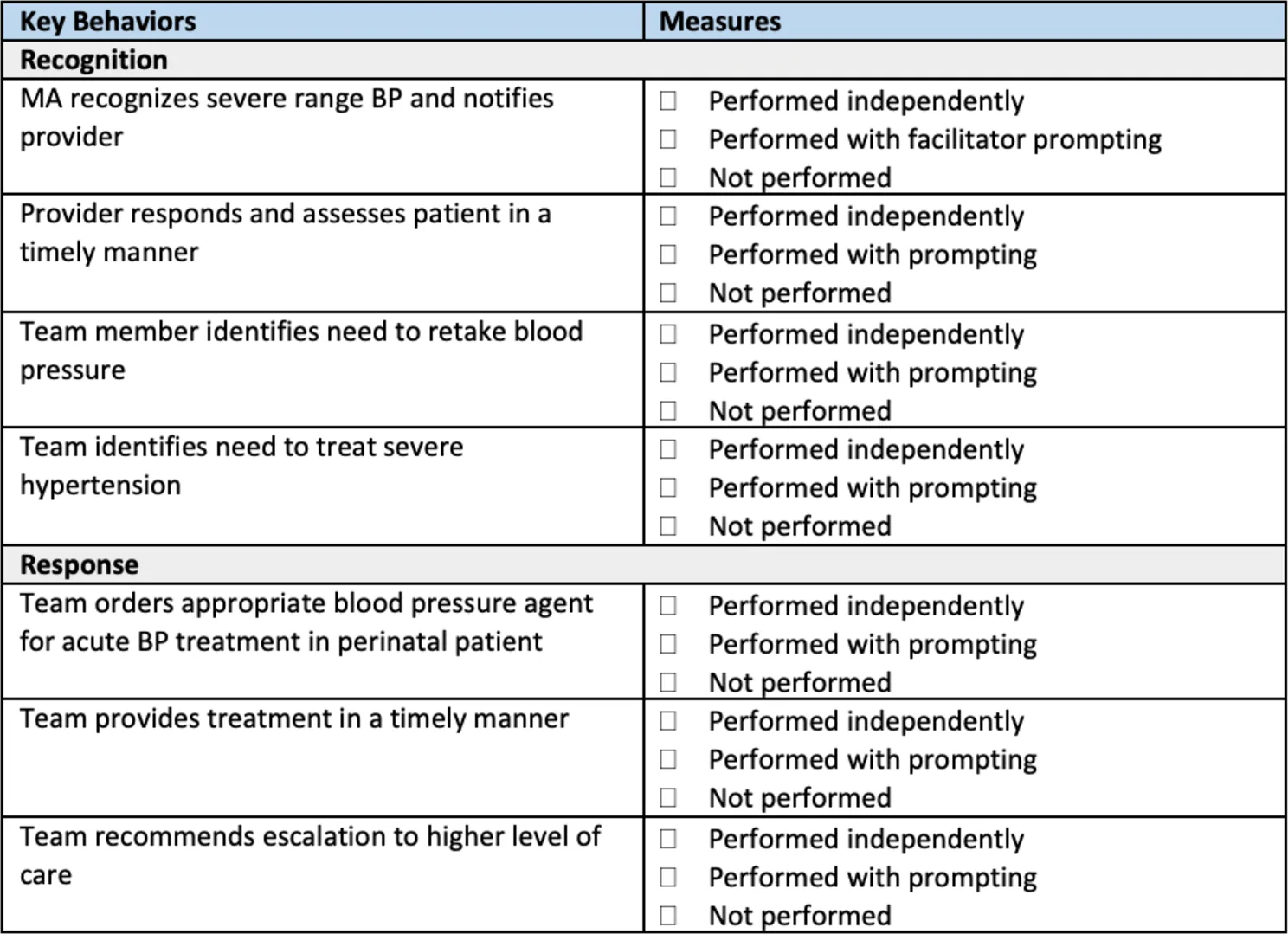
Simulation evaluation Form.”Performed independently” was scored as 2 points, “performed with prompting” 1 point, and “not performed” 0 points

To measure the primary outcome of fidelity to the O-HTN Algorithm, a Simulation Evaluation Form was completed by research team observers at each simulation (Simulation Evaluation Form, Fig. 3). The evaluation form included key behaviors demonstrating fidelity to the O-HTN Algorithm and thus appropriate recognition of and response to severe HTN in pregnancy. Teams received 2 points if they performed the algorithm-recommended behavior independently, 1 point if they performed the behavior with prompting, and 0 points if they did not perform the behavior when prompted. Scores for each team were totaled and divided by the total number of possible points and multiplied by 100% to obtain a composite recognition and response score.

Feasibility was measured as the proportion of prenatal care providers at each clinic who were able to participate in training and simulations. Acceptability was explored via a post-implementation Likert scale survey administered to clinical team members with a 5-point response scale that ranged from strongly disagree to strongly agree. The survey included statements such as:

1. The simulations were helpful to solidify knowledge learned in the training.
2. The training webinar would have been sufficient for my learning even without the simulations.
3. The training was held at a time that was practical for our team.
4. The simulations were held at a time that was practical for our team.

**Fig. 4:**
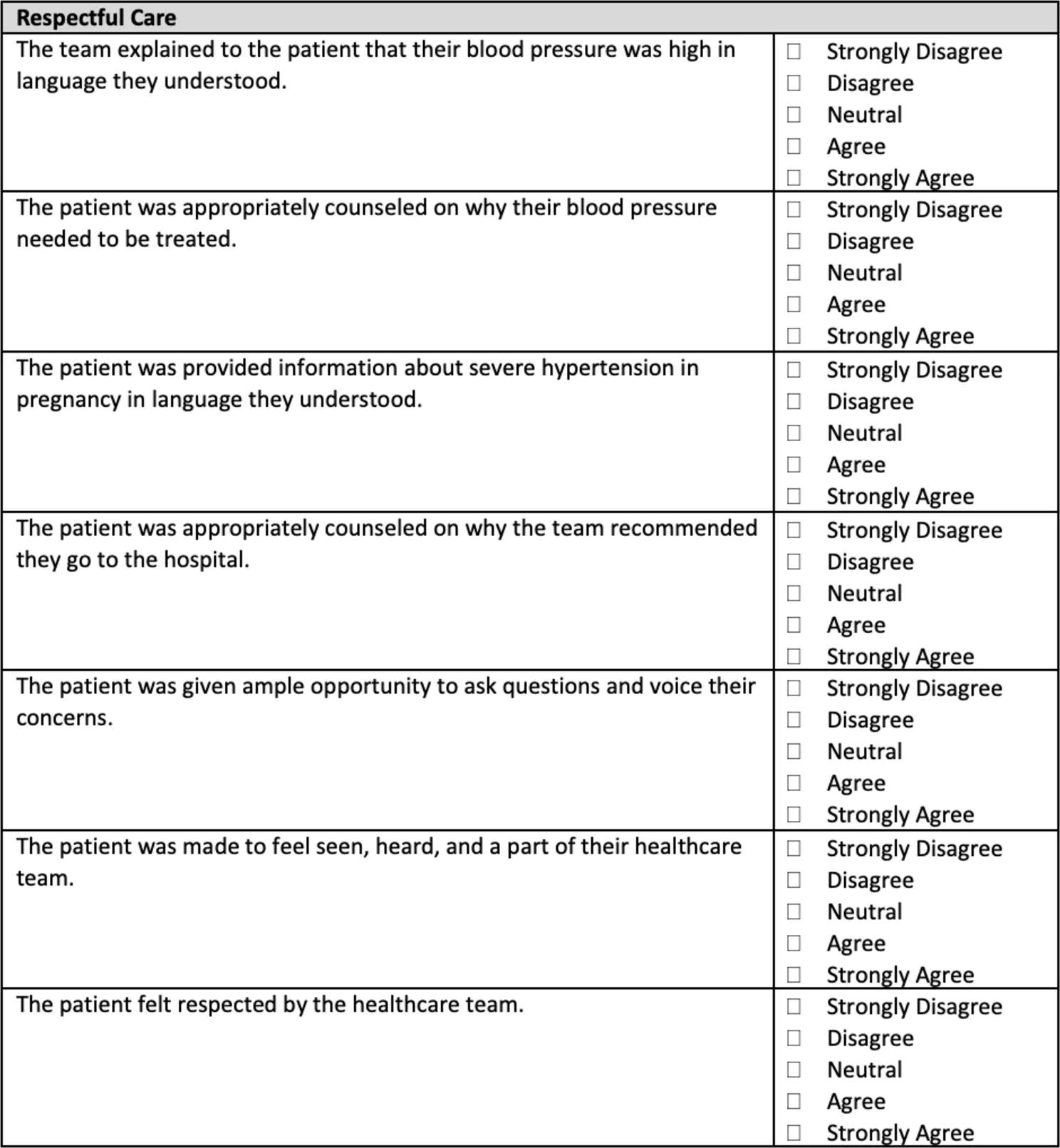
Respectful care reflection tool. Scoring: 0 points were assigned for response of strongly disagree, 1 for disagree, 2 for neutral, 3 for agree, and 4 for strongly agree. Points were then summed and divided by the total possible points (28) and multiplied by 100% to obtain a score

To measure the provision of respectful care, we used a Respectful Care Reflection Tool (RCR) developed through engaged partnership with local patients (Fig. 4). During simulation, teams were observed by research team members and evaluated using the RCR. Each clinical team member and the patient actor also completed the RCR for a 360° reflection. Teams received 0 points for response of strongly disagree, 1 for disagree, 2 for neutral, 3 for agree, and 4 for strongly agree. Points were then summed and divided by the total possible points (28) and multiplied by 100% to obtain a respectful care score.

### Analysis

Summary statistics, such as mean and standard deviation (SD), are reported for all measures. Simulation Evaluation and RCR scores were compared between pre- and post-training at the clinic level using a paired t-test, assuming the percentage of the possible points follows a normal distribution.

## Results

A total of 19 teams from three FHQCs participated in the study: 6 from Clinic 1, 7 from Clinic 2, and 6 from Clinic 3. The participating teams included 60% of prenatal care providers (physicians and midwives) at Clinic 1, 58% at Clinic 2, and 60% at Clinic 3. All 19 clinical teams (100%) participated in both pre-training and post-training simulations lasting approximately 20 minutes each. Eighteen of 19 providers (94.7%) who participated in the study attended either the live training session or watched the training webinar between the pre-training simulation and the post-training simulation.

**Fig. 5:**
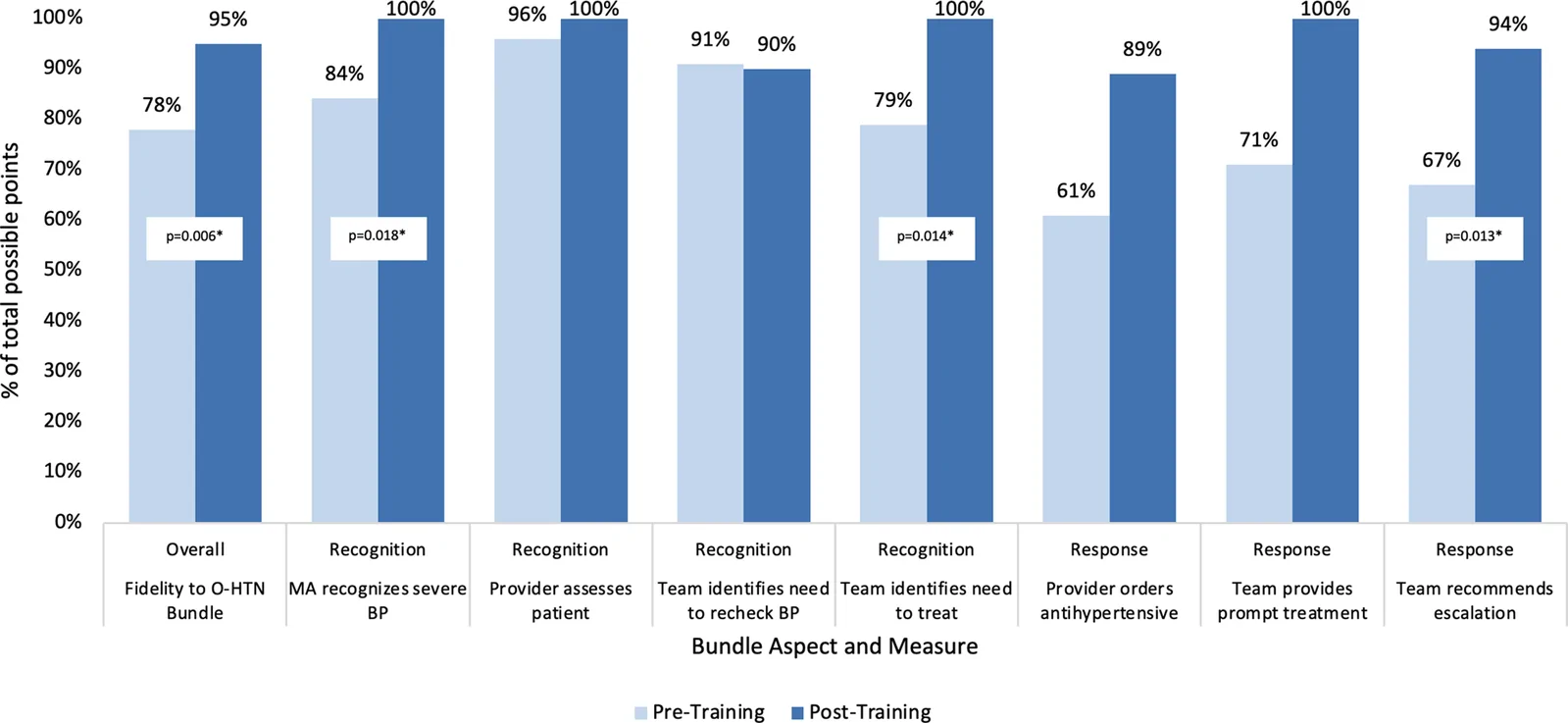
Fidelity to O-HTN bundle algorithm in pre- vs post-training simulations. *indicates statistically significant improvement

In the pre-training simulation, teams scored a mean of 78% [standard deviation (SD) 3%] overall on fidelity to the O-HTN Algorithm. Post-training, they improved to a mean of 95% (SD 2%, *p* = 0.006). Statistically significant gains were made on several key behaviors: identification of need to treat HTN (pre-training 79%, SD 4%, post-training 100%, SD 0%, *p* = 0.014), provision of timely treatment (pre-training 71%, SD 14%, post-training 100%, SD 0%, *p* = 0.075), and escalation to higher level of care (pre-training 67%, SD 8%, post-training 94%, SD 10%, *p* = 0.013) (Table 3, Fig. 5).

**Table 3:** Fidelity to O-HTN bundle algorithm in pre- versus post-training simulations^*^

Measure	Pre-Training average score (SD) *n* = 3^†^	Post-Training average score (SD) *n* = 3^†^	p-value
Overall fidelity to O-HTN Bundle	**78% (3%)**	**95% (2%)**	0.006
**Recognition of severe HTN**			
MA recognizes severe BP	84% (4%)	100% (0%)	0.018
Provider assesses patient	96% (7%)	100% (0%)	0.423
Team identifies need to recheck BP	91% (9%)	90% (9%)	0.985
Team identifies need to treat	**79% (4%)**	**100% (0%)**	0.014
**Response to severe HTN**			
Provider orders antihypertensive	61% (18%)	89% (10%)	0.069
Team provides prompt treatment	71% (14%)	100% (0%)	0.075
Team recommends escalation	**67% (7%)**	**94% (10%)**	0.013

* Scores presented as proportions of maximum possible points

† *n* = 3 clinics representing a total of 19 teams

**Fig. 6:**
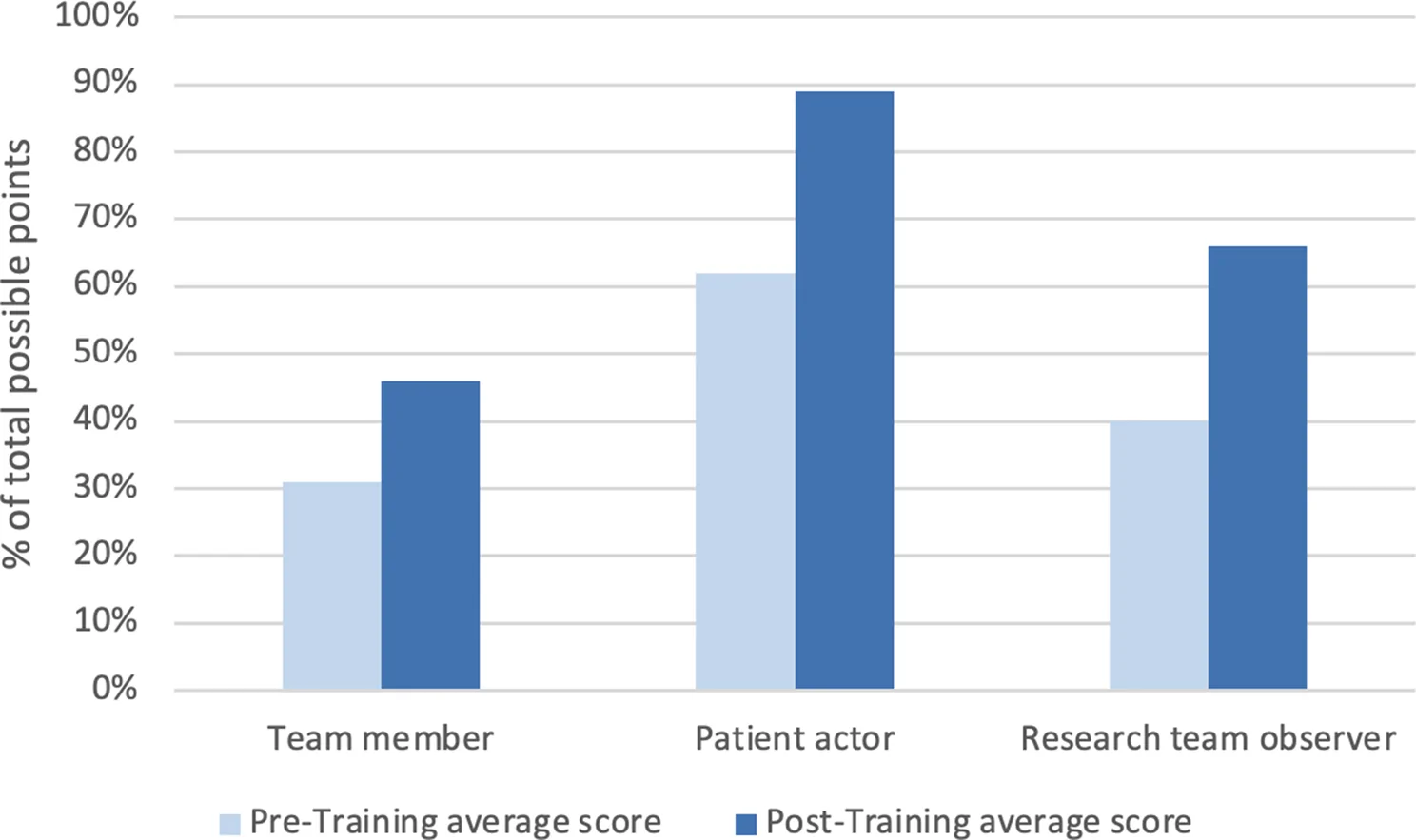
Respectful care reflection scores pre- versus post-training. *None of these improvements achieved statistical significance in this pilot study

Respectful care reflection scores improved after training for all groups, but improvements did not achieve statistical significance. In the pre-training simulation, clinical team members scored themselves an average of 31% (SD 10%) on the RCR compared to 47% (SD 12%) post-training (*p* = 0.114). The patient actor scored the teams an average of 57% (SD 27%) pre- and 88% (SD 4%) post-training (*p* = 0.168). The observer scored the teams an average of 39% (SD 8%) pre- and 68% (SD 11%) post-training (*p* = 0.102) (Table 4, Fig. 6).

**Table 4:** Respectful care reflection scores pre- versus Post-Training^*^

Evaluator	Pre-Training average score (SD) *n* = 3^†^	Post-Training average score (SD) *n* = 3^†^	p-value
Team member	31% (10%)	46% (12%)	0.114
Patient actor	62% (27%)	89% (4%)	0.168
Research team observer	40% (8%)	66% (11%)	0.102

* Scores presented as proportions of maximum possible points

† *n* = 3 clinics representing a total of 19 teams

Acceptability of the intervention was high (Table 5). Most post-implementation survey respondents (*n* = 28) agreed or strongly agreed that the training and simulations improved their team’s ability to recognize, treat, and escalate care appropriately for patients with severe hypertension in pregnancy (92.9%, 96.4%, and 96.4% respectively). The majority of respondents also agreed or strongly agreed that the training and simulations improved their team’s ability to provide respectful care to these patients (89.3%) and will change their practice (89.3%). A minority of respondents felt the training would have been sufficient without simulations (42.9%). The specific days and times chosen for the training and simulations were viewed as practical by only 60.7% and 50.0% of respondents respectively (Table 4).

**Table 5:**
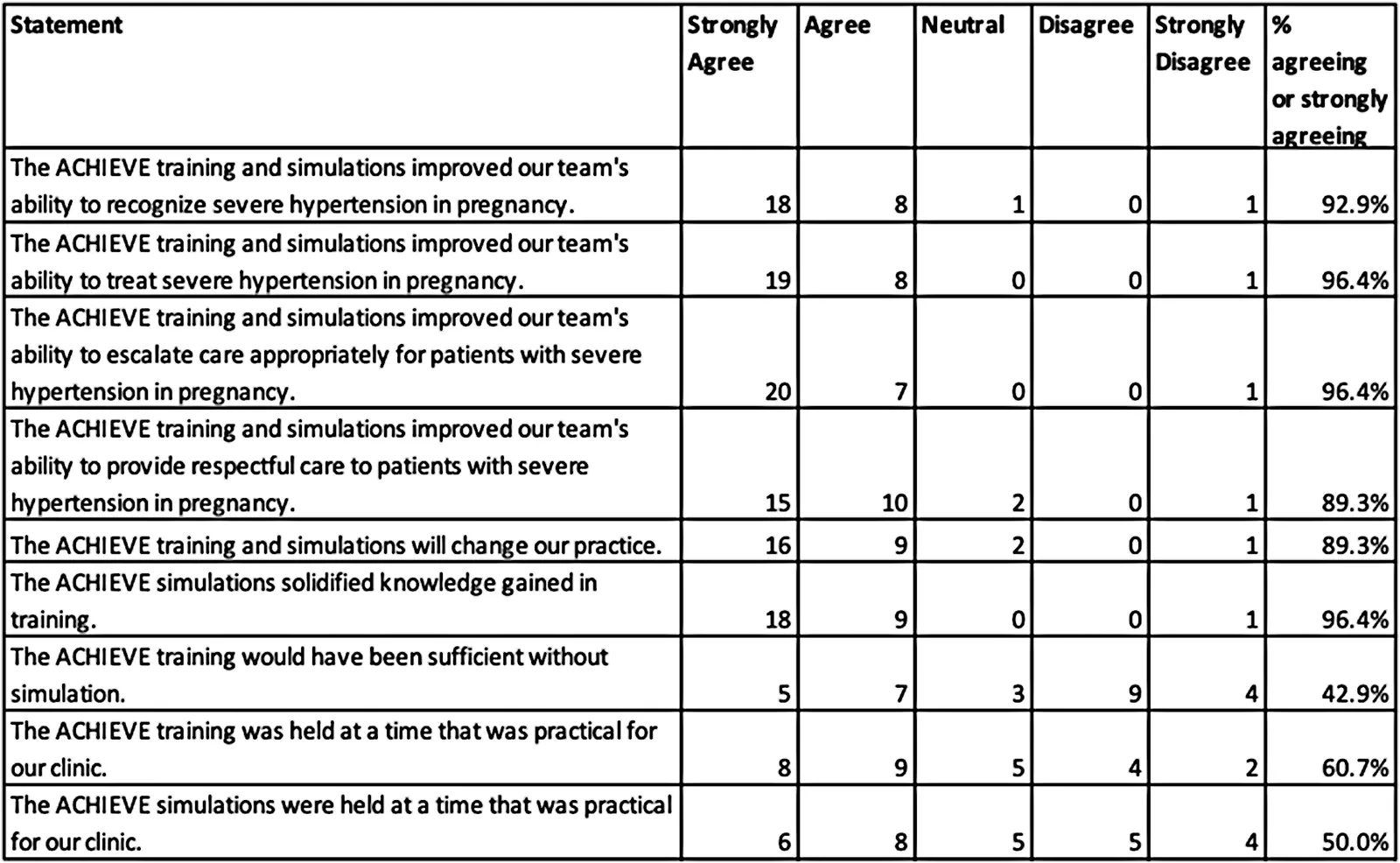
Post-implementation acceptability survey

Total respondents = 28

No harms or unintended effects were identified in this study.

## Structured discussion/comment

### Principal findings

In this pilot study, training and simulation were feasible, effective, and acceptable strategies for improving fidelity to the O-HTN Bundle in simulated episodes of severe hypertension in pregnancy. The strategies were used to improve respectful care provision in the simulated environment, but improvements did not achieve statistical significance in this pilot.

### Results

These results demonstrate the potential for improving recognition and response to severe hypertension in pregnancy through training and simulation in the outpatient setting are encouraging. While the AIM Severe HTN During Pregnancy and Postpartum Period Safety Bundle has been successfully implemented in hospitals and inpatient settings to decrease related maternal morbidity, to our knowledge its implementation has not been described in the outpatient setting. Furthermore, our findings add to the paucity of data on improving respectful care provision in the context of pregnancy.[26]

### Clinical implications

Training and simulation may be feasible and effective strategies for implementation of the Severe HTN During Pregnancy and Postpartum Safety Bundle in the outpatient setting. This is important as many episodes of severe HTN initially present in clinic rather than in the hospital, many times at sites where providers primarily care for individuals with low risk pregnancies. These are rare but critical events that require rapid recognition and response; effective simulation provides an opportunity to prepare and thus improve patient safety. The strategies assessed in this study have potential to support the implementation of guidelines for other rare but emergent events in the outpatient setting.

### Research implications

Given this was a pilot study in only three FQHCs, these implementation strategies will need to be further tested in a broader sample of clinics to fully assess their potential, including assessment of clinical team adherence to evidence-based guidelines when actual patients present with severe HTN in pregnancy pre- and post-implementation of the O-HTN Bundle rather than only in simulation. Additionally, future studies should assess the impact of training and simulation on the hypothesized causal mechanisms – care team knowledge, efficacy, and motivation. There is also need to incorporate implementation strategies that address additional elements of the O-HTN Bundle such as patient education. Further, we assessed fidelity to the O-HTN bundle at just one point, shortly after baseline assessment; in future studies, it would be important to assess fidelity at multiple points to assess stability of our findings and to determine whether fidelity is maintained over time.

### Strengths and limitations

This study was novel in that it used a community-engaged approach in collaboration with multiple FQHCs serving marginalized populations to collectively implement an innovative intervention for improving prenatal care of patients with hypertension. Nineteen individual obstetric care providers (including family medicine physicians and midwives) with their clinical team of nurses, medical assistants and pharmacists participated in training and simulations of an obstetric emergency often underrecognized and undertreated in the outpatient setting and improved their clinical capacity, demonstrating feasibility of this implementation strategy.

The study was limited by its pilot nature in that it only included 3 FQHCs, which impedes generalizability. It was also not specifically powered to an outcome. Additionally, there was potential for bias in the evaluation of simulations because observers from the research team knew whether the team was in their pre- or post-training simulation. Furthermore, it was not possible to assess or achieve inter-rater reliability as simulations were observed in real time by one member of the research team. Our measurement of respectful care was limited in that we did not have actual patients assessing it but rather standardized patients, providers, clinical staff, and research team observers. Also, we did not use validated measures for feasibility and acceptability and instead focused on more detailed assessments specific to the local context that would inform our future work. Lastly, we only evaluated recognition and response of severe HTN in simulations and do not have data on performance in situations of actual patients presenting with severe HTN. The next phase of this work will systematically address these limitations.

## Conclusions

Training and simulation hold promise as strategies for widespread implementation of an adapted outpatient severe HTN in pregnancy patient safety bundle. To further evaluate them, our team is currently leading a type 3 hybrid implementation-effectiveness study implementing the O-HTN Bundle in 20 clinics across North Carolina.[27]

## Electronic supplementary material

Below is the link to the electronic supplementary material.

Supplementary Material 1

## Data Availability

The datasets used and/or analyzed during the current study are available from the corresponding author on reasonable request.

## Abbreviations

US: United States
AIM: Alliance for Innovation on Maternal Health
HTN: Hypertension
O-HTN Bundle: Outpatient Hypertension Bundle
FQHC: Federally Qualified Health Center

## References

[1] Ford ND, Cox S, Ko JY, Ouyang L, Romero L, Colarusso T, et al. Hypertensive disorders in pregnancy and mortality at delivery hospitalization — United States, 2017–2019. MMWR Morb Mortal Wkly Rep. 2022;71(17):585–91. 10.15585/mmwr.mm7117a1. 35482575 PMC9098235

[2] Hitti J, Sienas L, Walker S, Benedetti TJ, Easterling T. Contribution of hypertension to severe maternal morbidity. Am J Obstet Gynecol. 2018;219(4):405.e1–e7. 10.1016/j.ajog.2018.07.002. 30012335

[3] Kuklina EV, Ayala C, Callaghan WM. Hypertensive disorders and severe obstetric morbidity in the United States. Obstet Gynecol. 2009;113(6):1299–306. 10.1097/AOG.0b013e3181a45b25. 19461426

[4] Fingar KR. Delivery hospitalizations involving preeclampsia and eclampsia, 2005-2014: Statistical brief #222. 2006. 28722848

[5] Coutinho T, Lamai O, Nerenberg K. Hypertensive disorders of pregnancy and cardiovascular diseases: current knowledge and future directions. Curr Treat Options Cardio Med. 2018;20(7):56. 10.1007/s11936-018-0653-8. 29923067

[6] Tooher J, Thornton C, Makris A, Ogle R, Korda A, Hennessy A. All hypertensive disorders of pregnancy increase the risk of future cardiovascular disease. Hypertension. 2017;70(4):798–803. 10.1161/HYPERTENSIONAHA.117.09246. 28893895

[7] Johnson JD, Louis JM. Does race or ethnicity play a role in the origin, pathophysiology, and outcomes of preeclampsia? An expert review of the literature. Am J Obstet Gynecol. 2022;226(2):S876–85. 10.1016/j.ajog.2020.07.038. 32717255

[8] Teal EN, Appiagyei A, Sheffield-Abdullah K, Manuck TA. Differences in disease severity and delivery gestational age between black and white patients with hypertensive disorders of pregnancy. Pregnancy Hypertens. 2022;28:88–93. 10.1016/j.preghy.2022.03.001. 35290940 PMC9724685

[9] Breathett K, Muhlestein D, Foraker R, Gulati M. Differences in preeclampsia rates between African American and Caucasian women: trends from the National Hospital Discharge Survey. J Educ Chang Women’s Health. 2014;23(11):886–93. 10.1089/jwh.2014.4749. 25211000

[10] Goodwin AA, Mercer BM. Does maternal race or ethnicity affect the expression of severe preeclampsia? Am J Obstet Gynecol. 2005;193(3):973–78. 10.1016/j.ajog.2005.05.047. 16157096

[11] Gestational hypertension and preeclampsia: ACOG practice bulletin, number 222. Obstet Gynecol. 2020;135(6):e237–60. 10.1097/AOG.0000000000003891. 32443079

[12] Martin JN Jr, Thigpen BD, Moore RC, Rose CH, Cushman J, May W. Stroke and severe preeclampsia and eclampsia: a paradigm shift focusing on systolic blood pressure. Obstet Gynecol. 2005;105(2):246–54. 10.1097/01.AOG.0000151116.84113.56. 15684147

[13] Judy AE, McCain CL, Lawton ES, Morton CH, Main EK, Druzin ML. Systolic hypertension, preeclampsia-related Mortality, and Stroke in California. Obstet Gynecol. 2019;133(6):1151–59. 10.1097/AOG.0000000000003290. 31135728

[14] Gupta M, Greene N, Kilpatrick SJ. Timely treatment of severe maternal hypertension and reduction in severe maternal morbidity. Pregnancy Hypertens. 2018;14:55–58. 10.1016/j.preghy.2018.07.010. 30527119

[15] Gynecologists AfIoMHACoOa. Severe hypertension in pregnancy patient safety Bundle. 2026. https://saferbirth.org/psbs/severe-hypertension-in-pregnancy/.

[16] Lee King PA, Young D, Borders AEB. A Framework to harness the power of quality collaboratives to improve perinatal outcomes. Clin Obstet Gynecol. 2019;62(3):606–20. 10.1097/GRF.0000000000000462. 31145112

[17] King P, Keenan-Devlin L, Gordon C, Goel S, Borders A. 4: reducing time to treatment for severe maternal hypertension through statewide quality improvement. Am J Obstet Gynecol. 2018;218(1):S4. 10.1016/j.ajog.2017.10.415. 38697335

[18] Shields LE, Wiesner S, Klein C, Pelletreau B, Hedriana HL. Early standardized treatment of critical blood pressure elevations is associated with a reduction in eclampsia and severe maternal morbidity. Am J Obstet Gynecol. 2017;216(4):415.e1–e5. 10.1016/j.ajog.2017.01.008. 28153655

[19] Miller CJ, Barnett ML, Baumann AA, Gutner CA, Wiltsey-Stirman S. The FRAME-IS: a framework for documenting modifications to implementation strategies in healthcare. Implementation Sci. 2021;16(1):36. 10.1186/s13012-021-01105-3. PMC8024675 33827716

[20] Lightfoot AF, FN, Healy J, Haidar J, Teal EN, Costello J, et al. Community-engaged, equity and implementation science-informed methods to adapt a severe hypertension in pregnancy and postpartum safety bundle and prepare for implementation in the outpatient setting. Under Review. 2026.

[21] Leeman J, Rohweder C, Lafata JE, et al. A streamlined approach to classifying and tailoring implementation strategies: recommendations to speed the translation of research to practice. Implement Sci Commun. 2024;5(1):65. 10.1186/s43058-024-00606-8. 38886763 PMC11181609

[22] Damschroder LJ, Reardon CM, Widerquist MAO, Lowery J. The updated consolidated framework for implementation research based on user feedback. Implement Sci. 2022;17(1):75. 10.1186/s13012-022-01245-0. 36309746 PMC9617234

[23] Kachoria AG, Fatima H, Lightfoot AF, et al. Understanding barriers and facilitators to implementation of a patient safety bundle for pregnancy-related severe hypertension in 3 North Carolina outpatient clinics: a qualitative study. Implement Sci Commun. 2025;6(1):7. 10.1186/s43058-024-00685-7. 39789591 PMC11715196

[24] Powell BJ, Waltz TJ, Chinman MJ, Damschroder LJ, Smith JL, Matthieu MM, et al. A refined compilation of implementation strategies: results from the expert recommendations for implementing change (ERIC) project. Implement Sci. 2015;10(1):21. 10.1186/s13012-015-0209-1. 25889199 PMC4328074

[25] Proctor EK, Powell BJ, McMillen JC. Implementation strategies: recommendations for specifying and reporting. Implement Sci. 2013;8(1):139. 10.1186/1748-5908-8-139. 24289295 PMC3882890

[26] Cantor AG, Jungbauer RM, Skelly AC, et al. Respectful maternity care: a systematic review. Ann Intern Med. 2024;177(1):50–64. 10.7326/M23-2676. 38163377

[27] Leeman J. Community-engaged implementation of a safety bundle for pregnancy-related severe hypertension in the outpatient setting: protocol for a type 3 hybrid study with a multiple baseline design. BMC Health Serv Res. 2024;24(1):1156. 10.1186/s12913-024-11579-8. 39350133 PMC11443898

