# Supplementary material for: Implementing an adapted patient safety bundle for pregnancy-related severe hypertension to improve recognition, response, and respectful care in the outpatient setting
Source: Implement Sci Commun. 2026 Jun 22;7:177. doi: 10.1186/s43058-026-01012-y (PMC13540961; doi:10.1186/s43058-026-01012-y)
Supplement: Supplementary file 1 [file 43058_2026_1012_MOESM1_ESM.pdf]

Pregnant Patient with Severe Hypertension in the Outpatient Setting
Total time: ~30 minutes (20 minutes for simulation /10 minutes for debrief)

Assigned Team Member Names: ____

Simulation Begins:
Facilitator reads scenario introduction:
The patient is a 39 y.o G3P1011 at 36 weeks gestation. She has a history of PCOS and A1GDM. Her A1GDM has been well controlled without medication. She has otherwise had an uncomplicated prenatal course and has no known drug allergies. She presents to her scheduled prenatal visit with no concerns. You may now begin rooming the patient and start your initial assessment.

Start Timer
Safe Performance and Team Communication checklist (15-20 minutes)

Participants should:	Prompts for facilitator:	Expected Behaviors:
Recognition<br>MA will correctly take BP and/or describe components of proper BP assessment	**Show Vital Signs after MA demonstrates or describes BP measurement**<br>BP: 165/108<br>HR: 80<br>O2 Sat: 100%<br>RR: 18	MA demonstrates or describes how to get correct fit/size of BP cuff<br>☐ Index line approach (index line must fit inside the two range lines)<br>☐ Measurement of arm circumference<br><br>MA describes components of proper BP Technique<br>☐ Empty bladder if needed<br>☐ Quiet room, sit quietly for 5 minutes<br>☐ Arm supported at heart level<br>☐ Legs uncrossed, feet on the ground<br>☐ Back supported<br>☐ Cuff on bare arm (no clothing)
Response<br>MA will notify provider of severe range BP<br><br>Provider responds to notification & comes to assess patient<br><br>MA or nurse repeats blood pressure	Patient does not have any of the severe symptoms– “I feel fine. I am just stressed because of the visit.”<br><br>**Repeat Vital Signs**<br>BP: 163/109<br>HR: 82<br>O2 Sat: 98%<br>RR: 18	MA notifies the provider:<br>☐ MA explains to patient that BP is high and that they are notifying the provider<br>☐ MA notifies provider via clinic-specified method of communication<br>☐ MA clearly specifies blood pressure<br><br>Provider responds promptly:<br>☐ Provider acknowledges elevated BP and responds to evaluate patient<br>☐ Provider explains the situation to the patient using language that the patient understands and respectful tone<br>☐ Provider assess patient for symptoms of severe pre-E (HA, vision changes, RUQ or epigastric pain)<br>☐ If resident physician, resident alerts preceptor and ask for additional help if needed<br>☐ MA, nurse or provider identifies need to retake BP<br>☐ MA or nurse demonstrates proper BP retake measurement and notifies provider

Provider rrecognizes need for treatment and escalation	State that 20 min have elapsed since treatment. Display new BP 145/94	Provider recognizes severe range BP and need for treatment and escalation<br>☐ Provider explains to patient that there is a severe range BP, why it is important to treat and recommends transfer for higher level of care<br>☐ Provider orders Nifedipine IR 10mg PO and communicates need for this medication to pharmacy<br>☐ Pharmacist dispenses Nifedipine<br>☐ Medication administered to patient by pharmacist, MA, RN or provider<br>☐ Provider notifies appropriate inpatient team to confirm the accepting facility<br>☐ RN calls EMS, arranges transport to hospital<br>☐ Provider addresses any patient or family questions or concerns, prepares them for potential hospital admission
	Case Conclusion:<br>EMS arrives and the patient is safely transported to the hospital. BP prior to leaving is 145/94. Pt remains asymptomatic. On arrival to the hospital, she remains stable and is admitted for pre-eclampsia w/ severe features by BP criteria.<br><br>The simulation is over. We will now review and debrief as a team	Scenario successful completed when team (all required):<br>☐ Recognizes and treats severe hypertension with one of an appropriate antihypertensive medication<br>☐ Diagnoses severe range BP and administration of antihypertensive medication.<br>☐ Counsels the patient regarding hypertensive disorder and need to escalate to higher level of care.

Team debrief (10 minutes) - if time is limited, focus on questions in **bold**

- What went well?
  - Communication: closed loop, care hand-off effectively?
  - Leadership: clear roles?
  - Mutual support: team helping w/ medication, speaking up, being an upstander?
- What did not go well? Use same framework as above.
- What could we do differently next time?
- **Assess readiness in debrief:**
  - **Was there rapid access to medication for treatment?**
  - **Was there a plan for escalation, obtaining appropriate consultation, and maternal transport (algorithm)?**
- **How did we provide care that was respectful to the patient and family? Were there gaps or areas of improvement in respectful care?**
  - **Engage patient actor here – what was their experience? How was the patient/family involved in their care and decision making?**
- Were there any biases that impacted the patient's care?
- How would this scenario have gone differently if the patient spoke Spanish?
- Did every member of the team have a voice in the exercise as well as the debrief?
